# A Buoyancy-Based Screen of Drosophila Larvae for Fat-Storage Mutants Reveals a Role for *Sir2* in Coupling Fat Storage to Nutrient Availability

**DOI:** 10.1371/journal.pgen.1001206

**Published:** 2010-11-11

**Authors:** Tânia Reis, Marc R. Van Gilst, Iswar K. Hariharan

**Affiliations:** 1 Department of Molecular and Cell Biology, University of California, Berkeley, California, United States of America; 2 Basic Sciences Department, Fred Hutchinson Cancer Research Center, Seattle, Washingon, United States of America; University of California San Francisco, United States of America

## Abstract

Obesity has a strong genetic component, but few of the genes that predispose to obesity are known. Genetic screens in invertebrates have the potential to identify genes and pathways that regulate the levels of stored fat, many of which are likely to be conserved in humans. To facilitate such screens, we have developed a simple buoyancy-based screening method for identifying mutant Drosophila larvae with increased levels of stored fat. Using this approach, we have identified 66 genes that when mutated increase organismal fat levels. Among these was a sirtuin family member, *Sir2*. Sirtuins regulate the storage and metabolism of carbohydrates and lipids by deacetylating key regulatory proteins. However, since mammalian sirtuins function in many tissues in different ways, it has been difficult to define their role in energy homeostasis accurately under normal feeding conditions. We show that knockdown of *Sir2* in the larval fat body results in increased fat levels. Moreover, using genetic mosaics, we demonstrate that *Sir2* restricts fat accumulation in individual cells of the fat body in a cell-autonomous manner. Consistent with this function, changes in the expression of metabolic enzymes in *Sir2* mutants point to a shift away from catabolism. Surprisingly, although *Sir2* is typically upregulated under conditions of starvation, *Sir2* mutant larvae survive better than wild type under conditions of amino-acid starvation as long as sugars are provided. Our findings point to a *Sir2*-mediated pathway that activates a catabolic response to amino-acid starvation irrespective of the sugar content of the diet.

## Author Summary

Obesity is a major problem in affluent societies. In addition to dietary intake, there are clearly genetic factors that make some people more likely to become obese. At present, we have a poor understanding of what the genetic differences are that predispose some individuals to obesity. In order to discover genes that regulate the amount of stored fat, we have conducted a study using larvae of the fruit fly Drosophila and shown that 66 different genes, when mutated, cause these larvae to store more fat. For the majority of these genes, very similar genes exist in humans. We have also shown that the Sir2 gene has a role in protecting these larvae from storing excessive amounts of fat and that it does so by regulating the synthesis and breakdown of fat in individual cells of a tissue where fat is stored. Finally, we demonstrate a role for Sir2 in changing metabolism when certain types of nutrients (amino acids) are lacking in the diet.

## Introduction

Obesity reflects an imbalance between the utilization and storage of energy, and involves a complex interplay between various tissues. At the cellular level, the pathways mediating the incorporation of circulating energy sources into intracellular storage forms such as triacylglycerides (TAGs) and glycogen are well understood, as are the pathways that convert stored energy into utilizable forms. In contrast, it is less clear how tissues balance these counteracting processes within an intact organism.

Drosophila larvae represent a promising model system for using a genetic approach to study the regulation of fat storage and utilization. The larval phase of development is dedicated to feeding, a behavior modulated by circuits in the brain. Ingested nutrients are used to synthesize cellular macromolecules required for the growth of larval tissues and for the growth and proliferation of cells of the imaginal discs, the precursors of adult structures such as the eye and the wing. In addition, energy is stored in a specialized organ called the fat body (FB), mostly as TAGs and glycogen. Fat stored in the FB can be broken down and utilized in other parts of the animal during the non-feeding pupal phase of development. Thus Drosophila larvae must have mechanisms that regulate the partitioning of ingested nutrients between storage and the generation of energy. The amount of fat stored in the larval FB is likely determined by regulation at the level of individual cells of the FB as well as endocrine and neuronal signals that involve other tissues.

Genetic screens for abnormalities in the mechanisms that regulate fat storage have been conducted in *C. elegans* and have demonstrated that a genetic approach in invertebrates can be used successfully to identify genes whose orthologs function in mammals to regulate fat storage [1], [2]. However, in contrast to the FB of Drosophila, which is an organ devoted primarily to energy storage, fat in *C. elegans* is stored in lipid droplets in intestinal epithelial cells. Thus screens that use Drosophila larvae have the added potential of uncovering pathways that are of relevance to the regulation of a tissue that is specialized for energy storage. Screens have been conducted in Drosophila tissue culture cells and Drosophila adults for increased fat stores [3], [4]. However, no screens of the larval stage have been reported to date.

In order to characterize mechanisms that regulate fat storage in the context of a growing organism, we designed a simple buoyancy-based screening strategy for identifying larvae that have increased levels of stored fat. Here we utilize this approach to identify 66 genes that potentially regulate organismal fat content, many of which have conserved mammalian orthologs. We also present the characterization of one of these genes, *Sir2,* in the tissue-specific regulation of fat levels, and demonstrate an unexpected survival advantage displayed by *Sir2* mutants under conditions of amino acid starvation.

## Results

### A buoyancy-based assay to identify mutant larvae with increased body fat levels

To identify genes that regulate the storage and utilization of energy at the level of the entire organism, we devised an indirect assay for body fat content in Drosophila larvae, based on the premise that individuals with a higher fat content float better in a solution of fixed density than lean individuals. This method is extremely rapid, inexpensive, and non-invasive, and enables the efficient screening of large numbers of animals. Importantly, following this type of non-invasive analysis, the larvae can be retrieved and either analyzed further or allowed to develop into viable and fertile adults.

**Figure 1:**
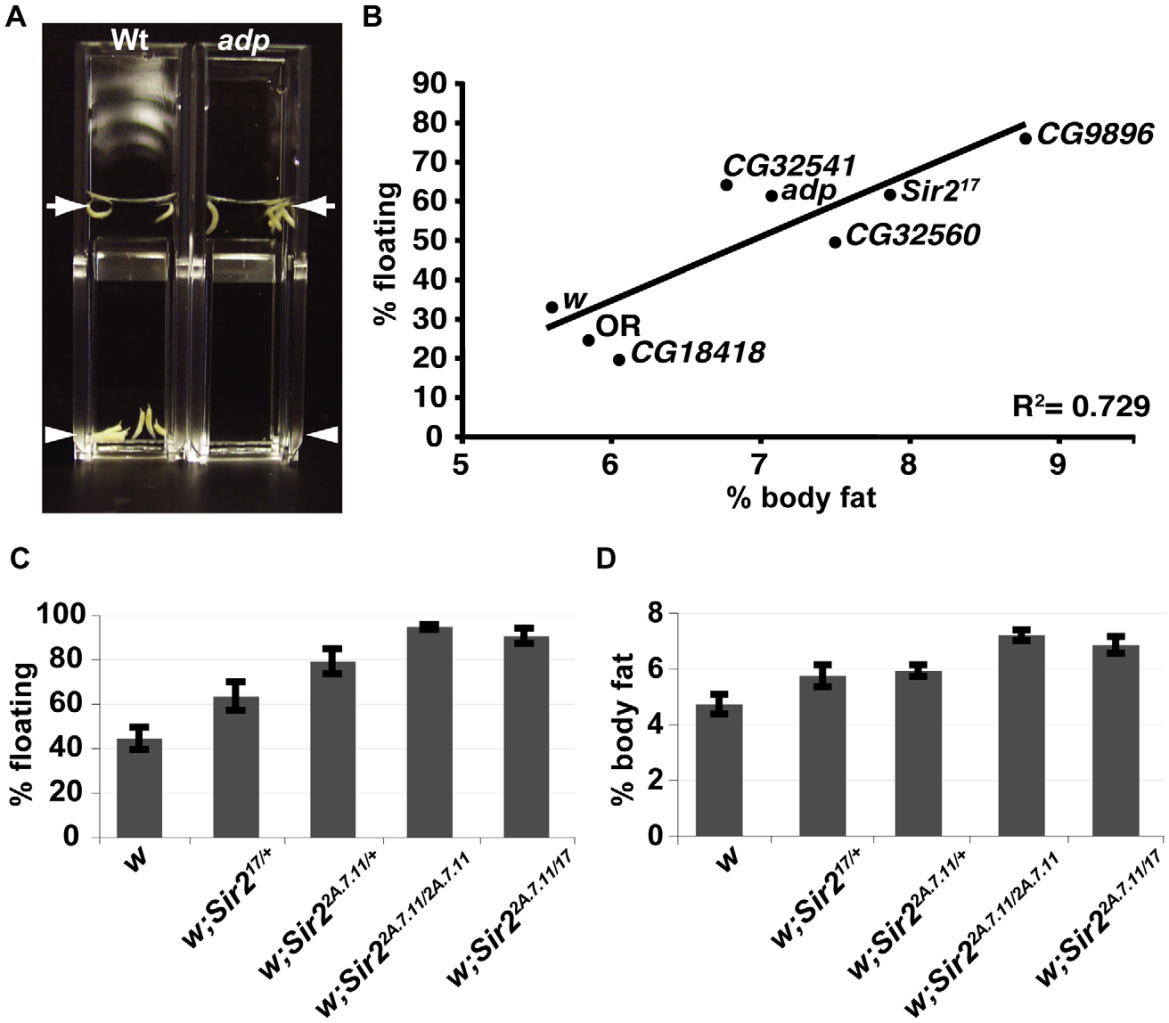
A buoyancy-based screen identifies a role for Sir2 in regulating fat levels in Drosophila larvae. (A) Wt or *adp* mutant larvae were immersed in the same concentration sucrose solution in plastic cuvettes and photographed after reaching equilibrium. *Arrows*, top of solution; *arrowheads*, bottom of solution. (B) For the indicated genotypes, mean floatation scores (% floating larvae; *y-*axis) were calculated from three independent biological replicates, for each using ∼50 larvae submerged in sucrose as in (A), and plotted against mean % body fat (*x*-axis) measured for triacylglycerides (TAG) by GC/MS and normalized to body weight (three independent biological replicates), as described in Materials and Methods. Oregon R (OR) is a wt control, *white* (*w*) and CG18418, a line from the collection that did not have a floating phenotype, are negative controls for the genetic background. The diagonal line shows the best-fit linear correlation (R^2^ = 0.73). (C) Flotation scores, determined for nine biological replicates of wt, heterozygous, or homozygous *Sir2* mutants as in Figure 1B. (D) % body fat, determined as in Figure 1B for five independent biological replicates. *Error bars* represent SEM.

When larvae were immersed in a ∼10% solution of sucrose, the majority of wild-type (wt) animals sink whereas *adipose* (*adp*) mutant larvae float (Figure 1A). *adp* is a conserved anti-obesity gene first identified as a naturally-occurring mutation in Drosophila [5], [6]. Equivalent results (not shown) were observed for the mutant *brummer*, which has increased fat levels. *brummer* is the ortholog of human adipocyte triglyceride lipase [7]. Conversely, larvae of the known lean mutant *lsd2* displayed a sinking phenotype in a lower-density sucrose solution in which wt larvae float (data not shown); *lsd2* encodes a protein with a perilipin-like PAT domain [8] and regulates formation of lipid droplets.

Using this technique, we screened a collection of ∼870 homozygous-viable mutants, comprising single transposon insertions in approximately 500 distinct genes [9]. For each mutant, the site of insertion has been mapped, thus simplifying the identification of the gene whose disruption most likely accounts for the mutant phenotype. After adding a 10% solution of sucrose in PBS to vials in which larvae had developed from eggs to the third larval instar stage, when they have the highest levels of stored fat, we selected mutant lines for which the majority of the larvae were floating. In addition, wild-type control (Oregon R) and positive control (*adp*) larvae were always assayed in parallel. Only those mutant lines with a reproducibly high percentage of floating larvae were analyzed further, with the exception of occasional lines with high scores in only a single test (Table 1).

**Table 1:** List of mutant Drosophila genes isolated in the floatation screen.

Mutant gene	Buoyancy score^a^	Molecular function^b^	Mammalian ortholog^b^
Oregon R^c^	–		
adpc	++	binding	Wdtc1
*CG32541*	+++	unknown	–
*NFAT*	+++	transcriptor factor activity	NFAT
*Fur1*	+++	serine-type endopeptidase activity	Furin
*CG6767*	+++	ribose phosphate diphosphokinase activity	Prps1
*Mod*	+++	chromatin binding	BACH1
*CG6854*	+++	CTP synthase activity	Ctps
*Arc1*	+++	nucleic acid binding	–
*trx*	+++	DNA binding	Mll1
*msn*	+++	protein serine/threonine kinase activity	Mink1
*Sip1*	+++	protein binding	Slc9a3r1
*jim*	+++	transcription factor activity	Mzf1
*trn*	+++	protein binding	Lrrn2
*CG11550*	+++	unknown	–
*CG1746*	+++	hydrogen-exporting ATPase activity	Atp5g2
*shep*	+++	mRNA binding	Rbms2
*4EHP*	+++	protein binding	Eif4e2
*neb*	+++	protein phosphatase 1 binding	Kif14
*CG32683*	+++	unknown	–
*CG3777*	+++	unknown	–
*grp*	+++	protein kinase activity	Chek1
*CG12587*	+++	unknown	–
*Glut1*	+++	glucose transmembrane transporter activity	–
*RpS23*	+++	structural constituent of ribosome	Rps23
*esg*	+++^ d^	RNA polymerase II transcription factor activity	Scrt2
*boi*	+++ d	unknown	Cdon
*dnt*	+++ d	transmembrane receptor protein tyrosine kinase activity	Ryk
*bgm*	+++ d	long-chain-fatty-acid-CoA ligase activity	Acsbg1
*eip75B*	+++	heme binding	Thra
*Dek*	+++ d	nucleic acid binding	DEK
*CG32699*	+++ d	acyltransferase activity	Lpcat1
*slim*	++ d	unknown	–
*CG32560*	++	Ras GTPase activator activity	–
*Fatp*	++ d	long-chain fatty acid transporter activity	Slc27a1
*cpo*	++	mRNA binding	–
*CG15309*	++	unknown	Ypel2
*Sir2*	++ d	NAD-dependent histone deacetylase activity	Sirt1
*Akt1*	++	protein serine/threonine kinase activity	Akt3
*pum*	++	mRNA binding	Pum1
*CG13791*	++	unknown	–
*CG1806*	++	unknown	–
Cerk	++	ceramide kinase activity	Cerk
*CG3774*	++	nucleotide-sugar transmembrane transporter activity	Slc35b4
*Gdi*	++	GDP-dissociation inhibitor activity	Gdi1
*CG14440*	++	unknown	–
*Fas2*	++	protein binding	Ncam2
*Vha16*	++	hydrogen-exporting ATPase activity, phosphorylative mechanism	Atp6v0c
*B-H2*	++ d	transcription factor activity	Barhl2
*psq*	++ d	DNA binding	–
*CG32556*	++ d	unknown	–
*chm*	++ d	transcription coactivator activity	Myst2
Sytβ	++ d	calcium-dependent phospholipid binding	–
*clt*	+	carboxylesterase activity	–
*or83b*	+^d^	protein binding	–
*nuf*	+	microtubule binding	Rab11fip4
*smi35A*	+	protein serine/threonine kinase activity	Dyrk4
*lilli*	+	transcription factor activity	Aff3
*h*	+	transcription repressor activity	H and E(spl)4
*CG13917*	+	protein binding	–
*CG10641*	+	calcium ion binding	Efhd2
*hdc*	+	unknown	Heca
*tmod*	+	actin binding	Tmod1
*jing*	+	transcription repressor activity	–
*CG33967*	+	protein binding	Wwc1
*CR32360*	+	tRNA	–
*tlk*	+	protein kinase activity	Tlk2
*l(1)G0007*	+	ATP-dependent RNA helicase activity	Dhx38

**a** Qualitative comparison of fraction of larvae floating in ∼10% sucrose.

**b** According to Flybase; blank means no ortholog predicted.

**c** Wild-type (Oregon R) and positive (*adp*) controls.

**d** Scored as floater in one experiment; insufficient animals due to sub-viability or developmental delay made the duplicate experiment unreliable.

Using this screening approach, we identified 66 genes that when mutated result in increased floatation, including a number of genes previously implicated in the regulation of fat storage in Drosophila or other organisms. For example, insertions in the olfactory receptor gene *or83b* caused floating phenotypes, consistent with reports that *or83b* adult Drosophila have increased TAG levels [10], thus demonstrating that this method is also capable of identifying non-cell-autonomous regulators of fat storage. The *cricklet* gene encodes a lipase expressed in the larval FB that is normally involved in the developmentally-induced breakdown of this tissue [11]; larvae lacking the *cricklet* gene product displayed floating phenotypes, which could be caused by the accumulation of extra fatty tissue. Akt is a kinase involved in insulin-mediated regulation of glucose and lipid metabolism, and adipose tissue accumulates in knockout mice with impaired Akt signaling [12]. Larvae with insertions in the Drosophila *akt1* gene also floated. Importantly, two thirds of the genes picked in our screen have predicted mammalian orthologs (Table 1), emphasizing the potential of this method to identify conserved regulators of body fat levels. Finally, in our screen we identified a line with a transposon insertion that disrupted the *Sir2* gene, which we chose to characterize further.

### Density and fat content of “floater” mutants are highly correlated

In order to determine whether the floating phenotype correlates with an increase in the levels of stored fat, eight “floater” mutants were chosen for more careful analysis. First, their floating phenotypes were quantified. Equivalent numbers of animals per vial were allowed to develop for 5 days to the wandering stage, at which point the floatation assay was performed. Developmental timing and crowding of cultures influences these results (data not shown). Hence, we only analyzed vials with 20–40 wandering (late third instar) larvae.

In order to measure levels of fat directly, we turned to gas chromatography/mass spectrometry (GC/MS), which allows an accurate quantitative and qualitative comparison of lipid content. From among the same animals scored for floatation (including floaters and sinkers), ten larvae per sample were chosen at random, and total neutral lipids were organically extracted. Neutral lipids derive primarily from stored TAGs, but also include circulating diacylglycerydes and free fatty acids. Values for percentage body fat represent the calculated total mass of all fatty acids (free and esterified) divided by the sample weight. All eight “floater” mutants selected for fat analysis by GC/MS displayed increased levels of body fat. The data for five of these are shown in Figure 1B. As previously reported [13], we find that in wt Drosophila, saturated C14 and saturated and monounsaturated C16 fatty acids predominate, with only traces of fatty acids longer than C18 (data not shown). The GC/MS profile indicated that for each of the eight mutants analyzed, there was a proportional increase in each fatty acid, showing that the mutations that were examined using this approach affect fat metabolism globally and not specific enzymatic reactions (e.g. fatty acid elongation). Moreover, when calculated as total neutral lipids per sample weight, percentage body fat correlated strongly with the floatation phenotype (Figure 1B) thus validating the use of buoyancy as a good indicator of organismal fat content.

### Mutation of the *Sir2* gene increases levels of stored fat in Drosophila larvae

The NAD-dependence of the sirtuin family of protein deacetylases couples cellular redox state to the acetylation state of sirtuin substrates, which include known regulators of glycolysis, gluconeogenesis, adipogenesis, and fatty acid oxidation, suggesting a central role for sirtuins in cellular energy homeostasis [14]. Moreover, in many species, including Drosophila, sirtuins appear to mediate the lifespan-extending effects of caloric restriction [15], implicating sirtuins in the physiological responses to the nutritional status of the organism. In addition to their short lifespan Drosophila *Sir2* mutants display a wide variety of apparently unrelated phenotypes, such as abnormalities in the physiological response to ethanol [16], defects in apoptosis [17], and a disruption of certain regions of heterochromatin [18]. However, a role in body fat regulation equivalent to that suggested for mammalian sirtuins has not been reported for Drosophila *Sir2* so far.

In our density-based screen we identified a line with a transposon insertion that is predicted to disrupt the *Sir2* gene. We therefore tested two independently-derived null alleles of *Sir2* and found that those mutants also had increased fat levels (Figure 1C). *Sir2^17^* lacks DNA sequences encoding the first 579 amino acids [18], whereas in the *Sir2^2a.7.11^* allele, all of the coding region is deleted [19]. Larvae heterozygous for *Sir2^17^* or *Sir2^2a.7.11^* scored consistently higher (less dense) than the wt control in the buoyancy assay (Figure 1C) and, by GC/MS, had ∼20% higher levels of body fat (Figure 1D). Animals homozygous for *Sir2^2a.7.11^* or trans-heterozygous for the two mutant *Sir2* alleles (*17/2a.7.11*) displayed even higher floatation scores (Figure 1C) and had ∼50% more fat than wt (Figure 1D).

### *Sir2* mutants eat less than wild-type larvae

Expression of the mammalian Sir2 homolog, SIRT1, is induced in the rodent hypothalamus upon fasting [20], [21], suggesting a possible neuronal role for sirtuins in energy homeostasis. Indeed, when fasted rats were fed, inhibition of SIRT1 function in the hypothalamus decreased their food intake and reduced the amount of weight they gained [21]. Paradoxically, SIRT1 expression has also been shown to increase in the hypothalamus upon feeding and its induction was hypothesized to play a role in satiety [22]. Consistent with previous studies that suggest that Sir2 is widely expressed [23], a GFP-expressing enhancer-trap line inserted near Drosophila *Sir2* is expressed in most, if not all, cells of every tissue examined, including the brain (Figure S1). To address the possibility that a behavioral defect in *Sir2* mutant larvae makes them eat more food and accumulate fat, we quantified rates of food consumption in *Sir2* mutant larvae. Following exposure to artificially-dyed food, developmentally-matched larvae were homogenized, a simple aqueous extraction was performed, and the amount of dye (directly related to the amount of food consumed) was quantified spectrophotometrically. *Sir2^2a.7.11^* mutants ingested ∼15% less food than their wt counterparts over a 30-min feeding interval (normalized proportional consumption ± standard deviation 0.85±0.12, *n* = 3; P = 0.0148 for paired two-tailed *t* test). Thus, the high-fat phenotype of *Sir2* mutants was not a result of increased food consumption. In addition, an RNA-mediated interference (RNAi) construct that clearly depleted endogenous *Sir2* (data not shown) had no detectable effect on feeding behavior when expressed specifically in neurons (Figure S2A) and knockdown of neuronal Sir2 caused a modest but reproducible “sinker” phenotype in the floatation assay (Figure S2B), accompanied by a slight decrease in stored fat (Figure S2C). These observations do not exclude the possibility that Drosophila *Sir2* may have an important function in specific neuronal circuits in regulating feeding behavior, since opposing functions for Sir2 in different sets of neurons would not be revealed if Sir2 levels were simultaneously reduced in all neurons. However, these observations suggest that the increased fat levels observed in *Sir2* mutants are not likely to result from a global loss of *Sir2* function in the brain. We therefore chose to examine the role of *Sir2* in the FB, an energy storage organ that performs some of the functions attributed to the liver and adipose tissue in mammals.

### Sir2 functions in the fat body to regulate organismal fat stores

**Figure 2:**
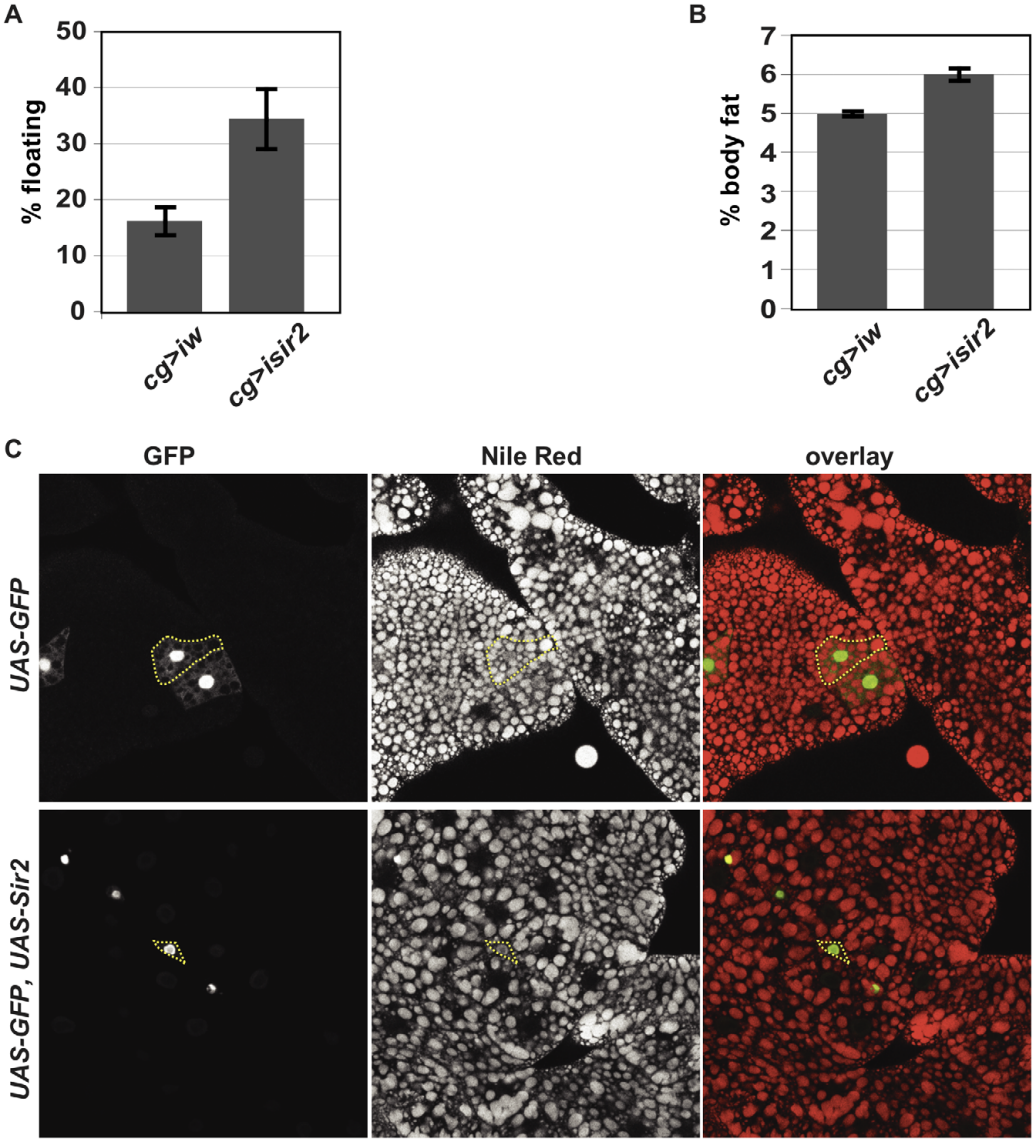
FB-specific manipulation of *Sir2:* knockdown increases organismal fat, whereas overexpression depletes lipid stores. (A) Floatation percentage and (B) %TAG per body weight of FB-specific *Sir2* depletion (“*cg>isir2*”) compared to control (“*cg>iw*”). Values represent averages of nine independent biological replicates for floating values and seven replicates for body fat; *error bars*, SEM. All lines are in the same genetic background, *w^1118^*. Floatation (data not shown) and %TAG for control *UAS-Sir2RNAi* animals lacking the Gal4 driver (5.6%±0.1%) were indistinguishable from Gal4-less *UAS-wRNAi* (5.6%±0.1%). (C) Larval FB tissue from animals ectopically expressing in clones of cells (*green*) GFP alone (*top row*) or GFP and *Sir2* (*bottom row*) generated by FLP-mediated recombination (as described in Materials and Methods), stained with the lipophilic dye Nile Red (*middle column*; *red* in *right column*). *Dashed yellow lines* outline single cells, as assessed by GFP fluorescence. Clones were obtained without induction of *flp*, relying on “leaky” *flp* expression during FB development.

Phenotypes elicited by tissue-specific SIRT1 manipulation have sometimes suggested seemingly contradictory functions for SIRT1 [24]–[26] (see Discussion). In any case, mammalian SIRT1 appears to regulate metabolic pathways in the liver, white adipose tissue, skeletal muscle and pancreatic beta cells [14]. To determine whether Sir2 regulates metabolic pathways in the cells of the FB, we depleted *Sir2* from the FB using RNAi. When we used the *cg-gal4* driver [27], [28], which is expressed at high levels in the FB, the resulting larvae had consistently higher floatation scores (lower density) than control larvae in which the interfering RNA was homologous to the *white (w)* gene (Figure 2A). A weaker flotation phenotype was observed using the *lsp-2* driver [29], which has weaker expression in the FB (data not shown). Thus, knockdown of *Sir2* in the FB appears to recapitulate the low-density phenotype of the *Sir2* mutants.

To determine whether these differences in larval density correspond to differences in total body fat content, the amount of neutral lipids was measured by GC/MS. Knockdown of *Sir2* using *cg-gal4* increased total body fat levels by approximately 20% (Figure 2B, mean ± SEM 6%±0.2% for *Sir2* RNAi compared to 5%±0.1% for *w* RNAi control; *p* = 0.01 for Paired *t-*test). The increase in fat content upon *Sir2* knockdown using *cg-gal4* (∼20%) was less severe than that observed for homozygous *Sir2* mutants (∼50%). This may result from residual *Sir2* activity in the FB following knockdown and/or contributions to body fat regulation by normal *Sir2* activity in other tissues. Food consumption was unaffected by *Sir2* knockdown using *cg-gal4* (data not shown), suggesting that Sir2 functions in the FB itself to regulate fat storage and metabolism.

### Excess Sir2 can function cell-autonomously in the FB to deplete fat stores

Our results point to a role for *Sir2* in the FB to restrict fat storage. If Sir2 functions by directly promoting catabolism of stored energy in the FB, then increasing Sir2 levels could reduce fat stores. To test this possibility, we generated mosaic animals in which cells overexpressing both *Sir2* and *GFP* were dispersed within an otherwise wild-type FB. The FB was stained with the lipophilic dye Nile Red to visualize lipid droplets and examined microscopically. Based on their morphology and the appearance of lipid droplets in those cells, control cells expressing GFP alone were indistinguishable from the surrounding wt cells (Figure 2C). In contrast, cells overexpressing *Sir2* were markedly smaller and contained fewer and smaller lipid droplets (Figure 2C), demonstrating that, even under conditions where nutrients were abundant, excess *Sir2* was sufficient to decrease fat stores in individual FB cells, despite the presence of surrounding wt cells. Thus, *Sir2* functions in a cell-autonomous manner to regulate fat stores in individual cells of the FB. This effect on the amount of fat stored in individual cells could result directly from regulation of fat metabolism by Sir2. Alternatively, *Sir2* overexpression could potentially reduce cell size and thereby indirectly prevent lipid droplet accumulation. Interestingly, consistent with an effect on depletion of energy stores, constitutive *Sir2* overexpression throughout the FB using the *cg-gal4* driver – but not constitutive pan-neuronal overexpression using *elav-gal4* – induced a developmental arrest and subsequent lethality (data not shown).

### Changes in expression of metabolic genes suggest a shift away from catabolism in the absence of *Sir2*

**Figure 3:**
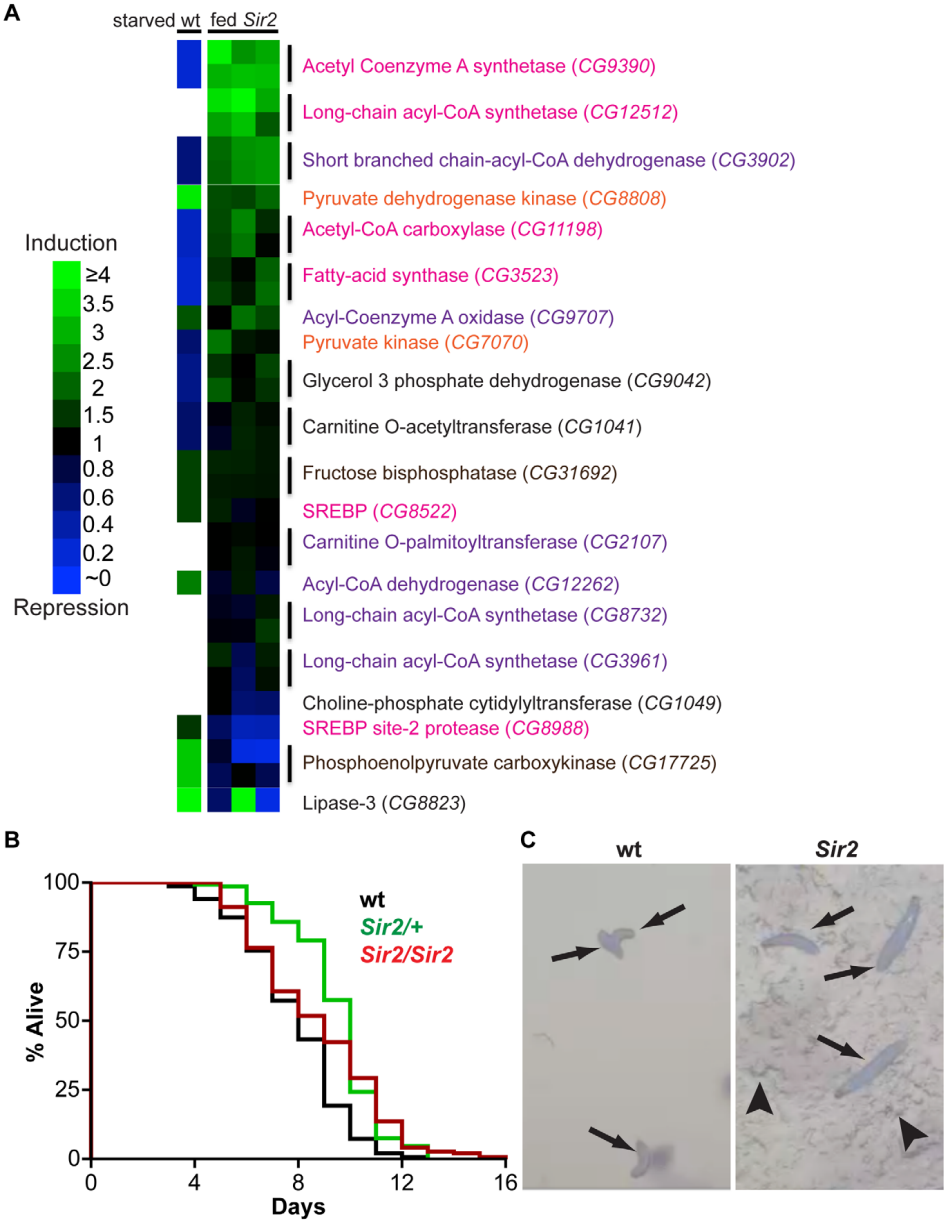
Starvation resistance and a shift in gene expression away from energy catabolism in *Sir2* mutants. (A) According to the legend at left, heat maps of changes in transcript levels of selected metabolic regulatory genes as detected by qPCR. “fed *Sir2*”, *Sir2* mutant larvae fed a standard diet, and compared to wt on the same diet, for three independent biological replicates (one replicate per column). “starved wt”, published expression changes [34] for starved wt larvae relative to fed. All larvae were at the third instar wandering stage. Text color corresponds to pathways shown in Figure S3. For *Sir2* samples, two independent primer sets (*vertical lines*) were used for most genes; for these genes in the starved wt sample, the same single value is shown in duplicate. (B) Larvae of the indicated genotypes were reared in amino acid-free media. Survival curves for larvae in a sucrose solution. For each genotype, three independent replicates of ∼50 larvae were pooled together after determining by Log-rank test that they were not significantly different (*P*>0.05). Differences between wt and the mutants were highly significant (Log-rank *P* values: wt *vs. Sir2/+*, <0.0001; wt *vs. Sir2/Sir2*, <0.0001; *Sir2/+ vs. Sir2/Sir2*, 0.27). (C) Larvae of the indicated genotype reared in a grape juice agar medium were photographed at day 12. *Arrows*, larvae; *arrowheads*, disruption of the agar surface due to larval feeding activity.

Since Sir2 can modulate the level of stored fat in individual cells, it is likely to operate via the regulation of cellular metabolism. Indeed, studies in cultured mammalian cells have identified several ways that SIRT1 influences cellular metabolism, mostly by regulating the transcription of key metabolic enzymes. SIRT1 directly deacetylates transcriptional regulators such as PGC-1alpha and FOXO1, resulting in increased levels of effectors of gluconeogenesis and fatty acid oxidation [14]. Also, deacetylation of SREBP, results in inhibition of lipid synthesis and fat storage [30]. Additionally, interaction of SIRT1 with cofactors of the nuclear receptor PPARγ activates the expression of genes that promote fat mobilization and represses genes required for fat storage [14]. SIRT1 is also found in the cytoplasm of some cells [31], where it can directly modify metabolic enzymes involved in lipogenesis, such as acetyl-coA synthetases [32]. Likewise, Drosophila Sir2 is predominantly nuclear but in certain situations exhibits cytoplasmic localization [33]. To determine whether gene expression changes are involved in the altered cellular metabolism of *Sir2* mutants, and to identify candidate downstream molecular targets of Sir2, we examined RNA levels of a panel of twenty key metabolic enzymes. When compared to developmentally-matched wt larvae, quantitative real-time reverse transcription PCR (qPCR) of mRNA from *Sir2* mutant larvae revealed pronounced alterations in the expression of 14 of 20 genes encoding components of glycolytic, gluconeogenic, fatty acid oxidation, and lipid processing pathways (Figure 3A and Figure S3). Two housekeeping genes, *actin5c* and *alpha-tubulin84B*, displayed little or no change.

Notably, in response to nutrient withdrawal, the levels of many of these same RNAs have been reported previously to undergo significant changes in wt larvae [34], but in directions opposite of those we observed under conditions of normal feeding for *Sir2* mutants. For example, RNA levels of the *CG9390* gene, encoding acetyl coenzyme A synthetase (AcCoAS), are repressed ∼5-fold in starved wt animals [34], but were ∼3-fold induced in *Sir2* mutants (Figure 3A). Similarly, phosphoenolpyruvate carboxykinase (PEPCK; *CG17725*) is elevated ∼3-fold in starved wt animals [34] but decreased ∼3-fold in *Sir2* mutants. AcCoAS catalyzes an important step in fatty acid synthesis (www.genome.jp/kegg/pathway.html), whereas PEPCK activity promotes gluconeogenesis (www.genome.jp/kegg/pathway.html). Accordingly, if the wt response to starvation represents a shift away from energy storage and towards mobilization, then the transcriptional changes we observed in *Sir2* mutants fed a standard diet appear to be biased towards energy storage/anabolism and away from energy mobilization/catabolism. *Sir2* is itself upregulated during starvation [34], suggesting that it likely functions in a pathway that mediates energy mobilization from the FB. Moreover, the changes in the levels of body fat and gene expression in fed *Sir2* mutants indicate that even on a standard diet Sir2 plays an important role in maintaining energy homeostasis.

### *Sir2* mutant larvae are resistant to amino acid starvation

Since Sir2 expression is upregulated during starvation, and the high-fat phenotype of *Sir2* mutants suggested a defect in mobilizing stored energy, it seemed likely that starvation would be particularly deleterious to *Sir2* mutants. When wt larvae are reared in the absence of amino acids, and provided with a source of sugar, they arrest development at the second instar and perish after ∼10 days [35]. During this starvation-like arrest, the FB undergoes gradual and visible diminution, suggesting depletion of fat stores. To examine the requirement for Sir2 in this process, *Sir2^2A.7.11^/Sir2^2A.7.11^* homozygotes, *Sir2^2A.7.11^*/+ heterozygotes, and wt animals were reared on a sugar-based medium that lacks amino acids. Surprisingly, *Sir2/Sir2* and *Sir2/+* larvae outlived wt larvae under these conditions (Figure 3B and 3C). Median survival in a sucrose solution was increased in *Sir2^2A.7.11^*/+ (10 days, *n* = 148 larvae) and *Sir2^2A.7.11^/Sir2^2A.7.11^* larvae (9 days, *n* = 147) relative to wt (8 days, *n* = 150), and the longest-surviving larvae were *Sir2^2A.7.11^/Sir2^2A.7.11^* homozygotes (four larvae survived past 13 days, a point at which all wt and heterozygous animals had perished; Figure 3B). The survival advantage conferred by *Sir2* mutation is all the more remarkable considering that, under normal conditions, *Sir2* mutants are short-lived and sensitive to various stressors [15], Notably, while FB tissue was visibly diminished in wt larvae at late time points, the FB persisted in mutant animals (data not shown), suggesting they maintained their fat stores during the time course of the experiment.

## Discussion

Using a simple screening method based on buoyancy, we have identified a number of genes that when mutated potentially increase the levels of stored fat in Drosophila larvae. From 870 homozygous-viable lines, representing ∼500 genes, we retained 66 lines. i.e. approximately 13% of lines tested. If a similar proportion of all Drosophila genes function as negative regulators of fat storage, we would expect that approximately 1000–2000 genes would score positive in this assay. This is higher than the number of genes (216) identified in a RNAi-based genome-wide screen that used a colorimetric assay for increased fat levels in adult Drosophila [4]. This might reflect the relative sensitivity of the two types of screening. Alternatively, a greater proportion of genes may regulate the levels of fat storage in the larval phase than in adults. One of the advantages of our screening method is that the stringency of the method can be altered easily by changing the density of the sucrose solution. For instance, a small decrease in density would result in the identification of fewer “floaters”. Importantly, at the density that we used, eight out of eight “floaters” tested had increased levels of stored fat, as directly measured by GC/MS. Moreover, the fat levels correlated with the “flotation score”. This implies that genes cannot be simply divided into those that affect the levels of stored fat and those that do not. Rather, these differences are quantitative and graded. Also, there was remarkably little overlap between our screen and the RNAi-based screen conducted in Drosophila adults. Only one gene (*Arc1*) was identified as a negative regulator of fat storage in both screens. Thus it is clear that multiple approaches, each with its advantages and “blind spots”, will perform complementary functions in identifying genes that regulate the levels of stored fat in Drosophila.

Our screen identified *Sir2* as a negative regulator of fat storage in Drosophila larvae. Although sirtuin function in mammalian energy homeostasis is a subject of intense research, it remains unclear how mammalian sirtuins operating in diverse tissues regulate metabolism at the organismal level. Sirtuin functions vary dramatically in different cell types, and transgenic mouse models often display complex phenotypes that vary even between seemingly identical experiments [26]. For example, while mammalian SIRT1 is thought to directly regulate adipogenesis in white adipose tissue [36] and gluconeogenesis and glycolysis in the liver, [37], [38] mice with liver-specific Sirt1 depletion had either higher body weight and more fat in liver cells [24], or lower body weight and less fat in white adipose tissues [25].

At least in Drosophila, most of the effects of a reduction in *Sir2* function on fat storage can be explained by a cell-autonomous function in the FB of Drosophila larvae. Thus this mode of regulation of fat levels by sirtuins may be evolutionarily more ancient. Organisms that have either evolved more complex ways of regulating fat storage in individual tissues or have linked environmental cues to fat storage in more sophisticated ways (e.g. anticipating food availability, impending hibernation) may have co-opted sirtuins to function in other tissues in novel ways.

Our studies have also uncovered an unexpected role for *Sir2* under conditions of amino-acid starvation. Under conditions of nutrient limitation, Drosophila larvae appear to sense the absence of amino acids and initiate a starvation response involving activation of a program of catabolism in the FB to generate energy for survival. This would eventually deplete their existing energy stores and result in their death. Mammalian SIRT1 is required for an analogous switch to catabolism upon nutrient deprivation [38]. If Drosophila Sir2 performs an equivalent function, *Sir2* mutant larvae might fail to undergo a catabolic switch under conditions of amino-acid starvation. Paradoxically, this might provide them with a survival advantage under artificial conditions in which amino acids are lacking but sugar is plentiful. Under these conditions, wt larvae may activate a catabolic program in the FB while *Sir2* mutants may be able to preserve stores in the FB and use the dietary sugar as their sole energy source. Indeed, the presence of sugar in the diet was necessary for the survival of *Sir2* larvae, since complete starvation resulted in lethality (data not shown). If this interpretation of this phenomenon is correct, it suggests that in wt larvae, a switch in utilization from dietary sources to stored energy occurs in response to amino acid starvation irrespective of the sugar content of the diet, and that *Sir2* functions in mediating this switch.

In summary, we have shown that a simple screening method based on buoyancy can be utilized to identify mutations that result in increased fat storage in Drosophila larvae. Our characterization of *Sir2* mutants shows that they accumulate excessive fat, that *Sir2* functions in the Drosophila fat body in a cell-autonomous manner to regulate fat storage, and that this occurs, at least in part, via the regulation of RNA levels of key metabolic enzymes. Finally, our studies implicate *Sir2* as a key regulator in the switch from utilizing ingested nutrients to stored fat as an energy source.

## Materials and Methods

### Fly strains and food

Oregon R, *w^111^* ^8^, *w*; *Sir2^17^*, *w*; *Sir2^2a.7.11^* and *w*; *elav-gal4* were obtained from the Bloomington stock center. Other lines have been reported elsewhere: *w*; *cg-gal4* [27], *w*, UAS-Sir2 RNAi from the Vienna Drosophila RNAi Center [39], *adp* [5], [40], and *w*; UAS-Sir2 [17], *w*; *act>cd2>gal4* UAS-GFP and *yw hs flp^122^* [41].

Animals were reared at 25°C on a modified Bloomington media (with malt) containing 35 g yeast per liter. Food was made fresh every week and used within 2 weeks.

For all experiments, eggs were collected on grape plates at 25°C and 24 hr later 50 first-instar larvae were transferred to each vial.

### Density assay

∼50 animals per vial were allowed to develop for 5 days to the wandering stage before adding 10 ml of 10% sucrose (Fisher Scientific) dissolved in PBS. Developmental timing influences these results; hence, analysis was limited to vials with 20–40 wandering larvae. After gentle mixing and several minutes without agitation to achieve equilibrium, we counted the number of larvae floating at the surface. The total number of larvae in the vial was then determined by slowly adding 20% sucrose until all animals floated.

### Screen

The floatation assay was performed to screen a collection of ∼870 homozygous-viable mutants, each containing a single transposon insertion [9]. For each mutant, the site of insertion has been mapped, allowing easy identification of the gene most likely to be responsible for the mutant phenotype. Briefly, adult flies in vials were allowed to lay eggs overnight, whereupon the adults were removed and eggs were allowed to develop until the wandering larval stage. A 10% solution of sucrose (Fisher Scientific) in PBS was added to each vial and, after allowing the animals to reach density equilibrium, mutant lines for which the majority of the larvae were floating were selected. Wild-type control (Oregon R) and positive control (*adp*) larvae were always cultivated and assayed in parallel. In each screening session, ∼100–200 lines were screened in duplicate or triplicate, and only those with reproducibly high floatation scores were analyzed further, with the exception of occasional lines with high scores in only a single test.

### Lipid extraction and GC/MS

Ten larvae from among the total in each vial (including floaters and sinkers) were chosen at random and frozen in liquid nitrogen. These were weighed as a group, homogenized with a motorized pestle, and neutral lipids were extracted and analyzed by GC/MS as previously described [42]. Values for percentage body fat represent the total of all fatty acids divided by the sample weight.

### Feeding assay

Twenty five larvae (90–96 hr AED) per sample were added to a spot of yeast paste containing 0.5% food coloring FD&C Red #40 (Spectrum) in the center of a grape plate. Following 30 min at 25°C, twenty larvae per sample were washed and homogenized in 900 µl PBS. The absorbance at 520 nm of the aqueous phase was measured with a Spectronic Genesys 5 spectrophotometer. Importantly, as with control animals, all experimental larvae (*i.e*., mutant or expressing RNAi constructs) were found in or on the yeast paste (unpublished observations), demonstrating that differences in dye uptake were not the result of consumption of the grape juice agar instead of the dyed food.

### Mosaic analysis

Wandering third-instar larvae of the genotypes *hs flp^122^*; *act>cd2>gal4* UAS-GFP or *hs flp^122^*; *act>cd2>gal4* UAS-GFP UAS-Sir2 were dissected in PBS and fixed 1 hr @RT in 8% paraformaldehyde (Electron Microscopy Sciences)/PBS. Carcasses were stained at RT for 30 min with 62.5 ng/ml Nile Red (Invitrogen) dissolved in PBS.

### RT-qPCR

For each sample, RNA was extracted from 10 larvae using Trizol (Invitrogen) according to the manufacturer's instructions. RNA was purified using the RNaesy kit (Qiagen). RT was performed using Oligo d(T) 23 VN (NEB) and M-MuLV Reverse transcriptase (NEB) per manufacturer's instructions. qPCR was performed using SYBR GreenER qPCR Super Mix (Invitrogen), and primer sets were calibrated using serial dilutions of cDNA. Reactions were run in an Applied Biosystems Step One Plus qPCR machine. For each experiment, three independent biological replicates were performed. When possible, two independent primer sets were used per target. Primer sequences, available upon request, were designed to amplify the 3′ end of mRNA and span introns when possible. qPCR data were normalized to an average of the levels of *actin5c*, *alpha-tubulin84B*, *dhr3*, *cg5321* and *cg12703*.

### Starvation resistance

For each sample, 50 larvae were placed in a 20% sucrose/PBS solution and daily analyzed for viability by response to physical prodding. Dead larvae were removed immediately after scoring and the medium was changed daily. Prism 4 was used for statistical analysis and generation of survival curves.

## Supporting Information

Figure S1 Expression of Drosophila *Sir2* in larval tissues. An enhancer-trap line in which *GFP* is expressed from the *Sir2* locus [Kelso, et al] was used to examine Sir2 expression patterns in larval tissues. Tissues were dissected and stained with the indicated reagents before examination by fluorescence microscopy. *Green*, GFP fluorescence. (A) Brain; *yellow*, overlap of staining with anti-Elav antibodies and GFP fluorescence. (B) Fat body. (C) Gut. (D) Salivary gland; *blue*, DAPI (DNA). Note that the restricted subcellular localization of GFP in certain cell types suggests the *GFP* insertion at the *Sir2* locus encodes a fusion of GFP with non-GFP sequences that influence its localization. [Kelso RJ, Buszczak M, Quinones AT, Castiblanco C, Mazzalupo S, et al. (2004) Flytrap, a database documenting a GFP protein-trap insertion screen in Drosophila melanogaster. Nucleic Acids Res 32: D418-420.
(2.48 MB TIF)

Figure S2 Neuron-specific *Sir2* depletion does not affect food intake but decreases organismal fat levels. (A) Quantity of food ingested (absorbance) per 20 larvae of neuronal-specific *Sir2* depletion when compared to control. Values represent averages of three independent biological replicates; *error bars*, standard deviation. (B) Decrease in larval buoyancy upon brain-specific depletion of Sir2. (C) Decrease in % TAG per body weight upon brain-specific Sir2 depletion. Values represent averages of nine independent biological replicates for floating values and seven replicates for body fat; *error bars*, SEM. All lines are in the same genetic background, *w^1118^*.
(0.18 MB TIF)

Figure S3 Selected elements of putative Drosophila fatty acid synthesis, fatty acid oxidation, glycolysis and gluconeogenesis pathways. Genes whose expression was examined were assigned to particular reactions (*solid arrows*) according to the Kegg Pathway Database (www.genome.jp/kegg/pathway.html). Arrows on both ends indicate reversible reactions. Dashed arrows pointing to genes indicate activation of that gene's expression or enzymatic function; bar-headed lines indicate inhibition.
(0.46 MB TIF)
