# Supplementary figures and images for: A Buoyancy-Based Screen of Drosophila Larvae for Fat-Storage Mutants Reveals a Role for Sir2 in Coupling Fat Storage to Nutrient Availability
Source: PLoS Genet. 2010 Nov 11;6(11):e1001206. doi: 10.1371/journal.pgen.1001206 (PMC2978688; doi:10.1371/journal.pgen.1001206)

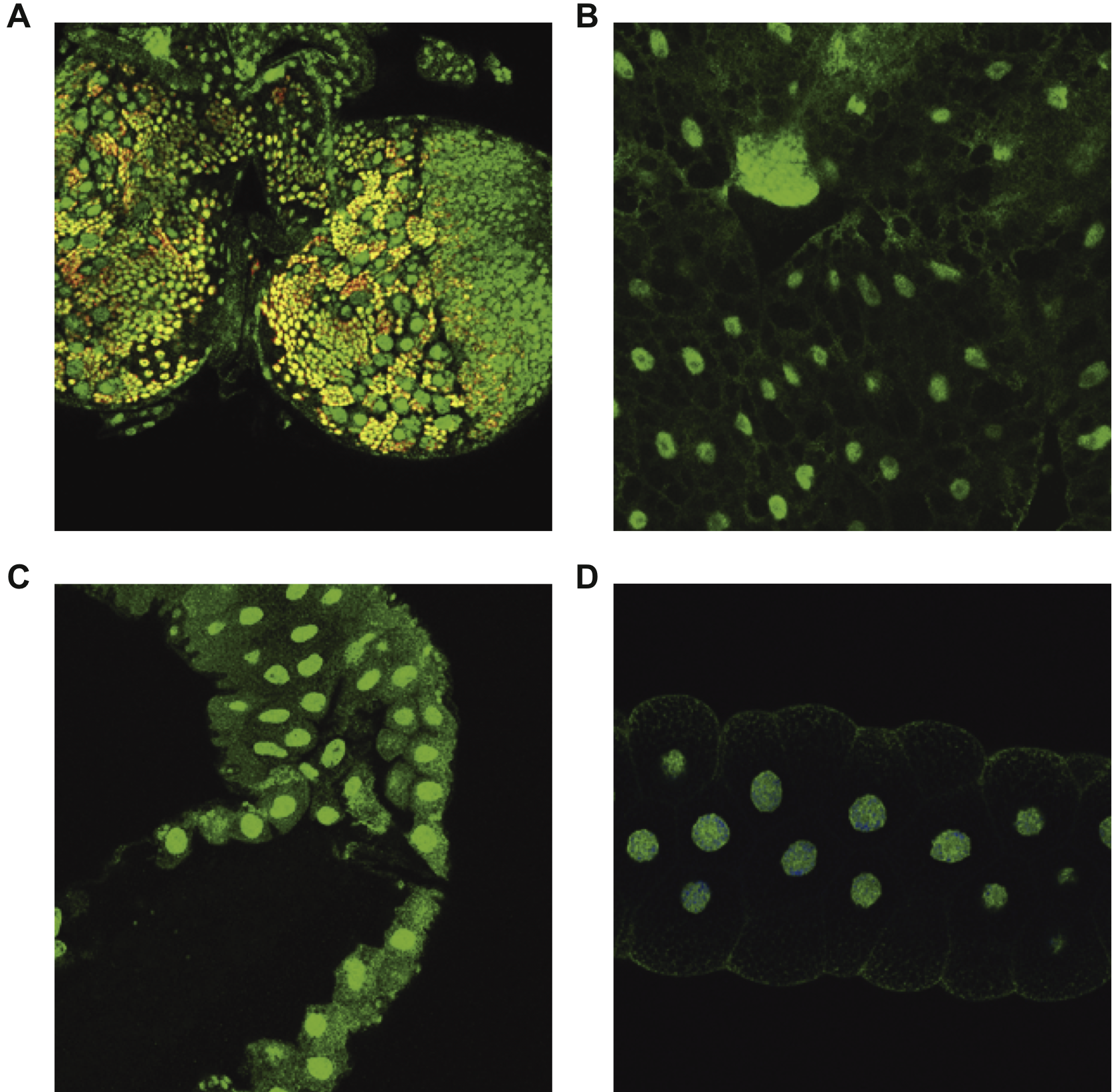

Supplement: Figure S1 — Expression of Drosophila Sir2 in larval tissues. An enhancer-trap line in which GFP is expressed from the Sir2 locus [Kelso, et al] was used to examine Sir2 expression patterns in larval tissues. Tissues were dissected and stained with the indicated reagents before examination by fluorescence microscopy. Green, GFP fluorescence. (A) Brain; yellow, overlap of staining with anti-Elav antibodies and GFP fluorescence. (B) Fat body. (C) Gut. (D) Salivary gland; blue, DAPI (DNA). Note that the restricted subcellular localization of GFP in certain cell types suggests the GFP insertion at the Sir2 locus encodes a fusion of GFP with non-GFP sequences that influence its localization. [Kelso RJ, Buszczak M, Quinones AT, Castiblanco C, Mazzalupo S, et al. (2004) Flytrap, a database documenting a GFP protein-trap insertion screen in Drosophila melanogaster. Nucleic Acids Res 32: D418-420. (2.48 MB TIF) [file pgen.1001206.s001.tif]

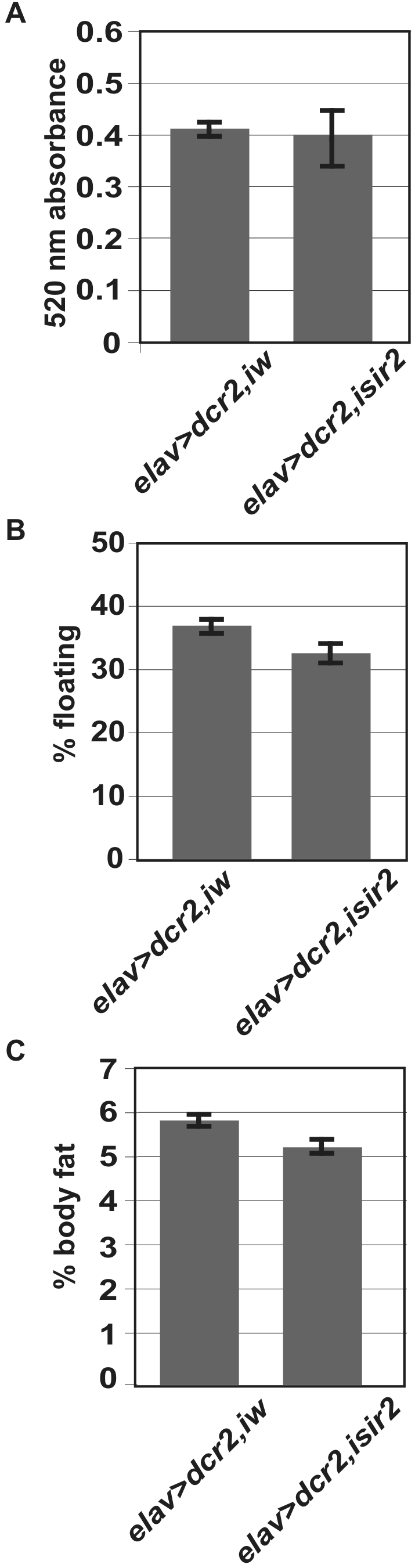

Supplement: Figure S2 — Neuron-specific Sir2 depletion does not affect food intake but decreases organismal fat levels. (A) Quantity of food ingested (absorbance) per 20 larvae of neuronal-specific Sir2 depletion when compared to control. Values represent averages of three independent biological replicates; error bars, standard deviation. (B) Decrease in larval buoyancy upon brain-specific depletion of Sir2. (C) Decrease in % TAG per body weight upon brain-specific Sir2 depletion. Values represent averages of nine independent biological replicates for floating values and seven replicates for body fat; error bars, SEM. All lines are in the same genetic background, w1118. (0.18 MB TIF) [file pgen.1001206.s002.tif]

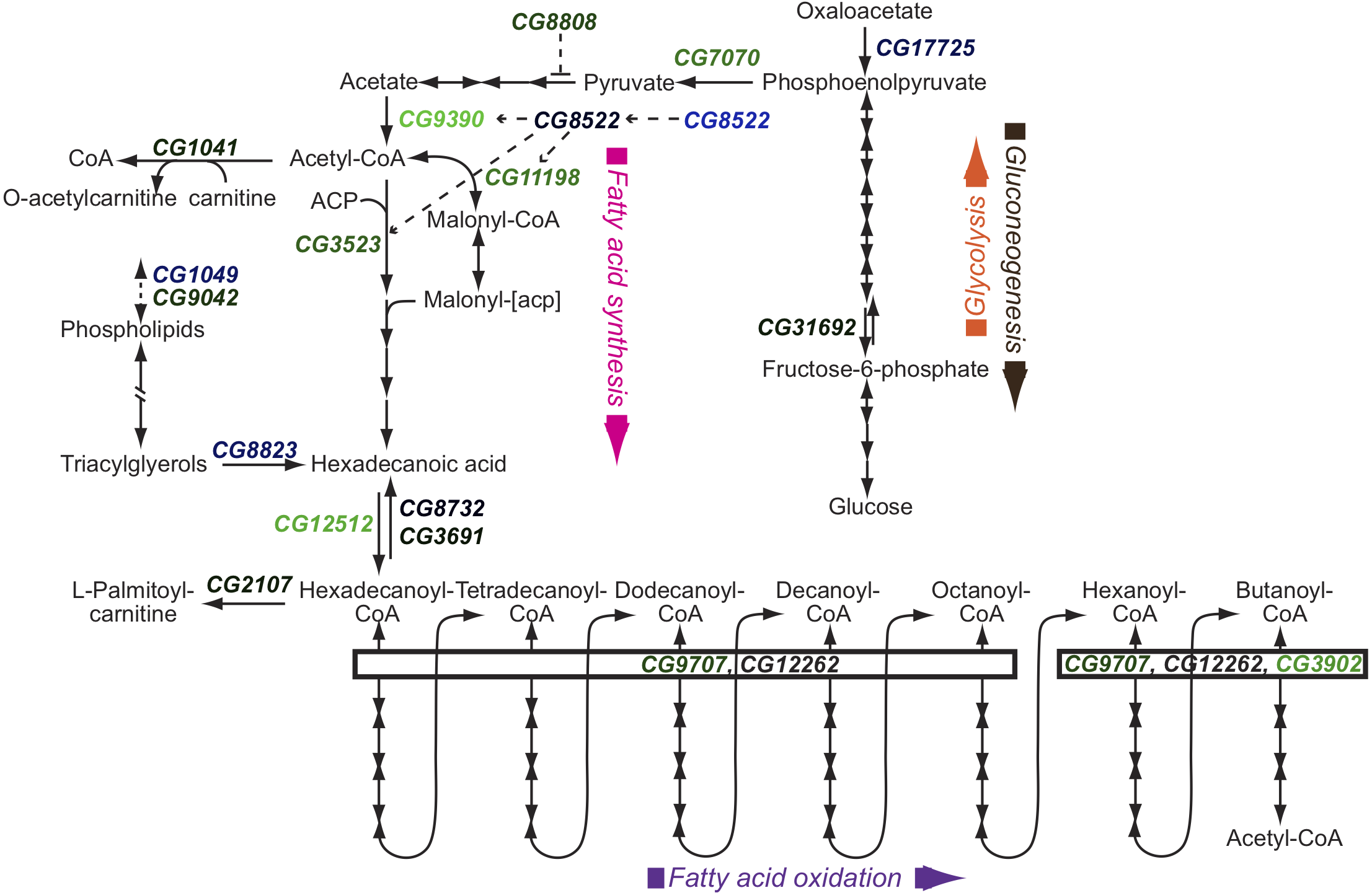

Supplement: Figure S3 — Selected elements of putative Drosophila fatty acid synthesis, fatty acid oxidation, glycolysis and gluconeogenesis pathways. Genes whose expression was examined were assigned to particular reactions (solid arrows) according to the Kegg Pathway Database (www.genome.jp/kegg/pathway.html). Arrows on both ends indicate reversible reactions. Dashed arrows pointing to genes indicate activation of that gene's expression or enzymatic function; bar-headed lines indicate inhibition. (0.46 MB TIF) [file pgen.1001206.s003.tif]
